# Statement of Retraction: CRISPR/Cas9 (D10A) nickase-mediated Hb CS gene editing and genetically modified fibroblast identification

**DOI:** 10.1080/21655979.2026.2729951

**Published:** 2026-10-01

We, the Editors and Publisher of the journal Bioengineered, have retracted the following article from publication.

Wu, W. H., Ma, X. M., Huang, J. Q., Lai, Q., Jiang, F. N., Zou, C. Y., Chen, L. T., & Yu, L. (2022). CRISPR/Cas9 (D10A) nickase-mediated Hb CS gene editing and genetically modified fibroblast identification. *Bioengineered*, 13(5), 13398–13406. https://doi.org/10.1080/21655979.2022.2069940  

Since publication, significant concerns have been raised about the integrity of the data and reported results in the article.

When approached for an explanation, the authors answered our queries, but the responses did not sufficiently address the concerns, and they were not able to provide the original data or supporting documentation.  

As verifying the validity of published work is core to the integrity of the scholarly record, we are therefore retracting the article. The authors have been informed of this decision, and agree with the retraction.

We have been informed in our decision-making by our policy on publishing ethics and integrity and COPE guidelines.  

The retracted article will remain online to maintain the scholarly record and will be digitally watermarked on each page as ‘Retracted’.  

